# Biological Basis of Tumor Angiogenesis and Therapeutic Intervention: Past, Present, and Future

**DOI:** 10.3390/ijms19061655

**Published:** 2018-06-04

**Authors:** Girolamo Ranieri

**Affiliations:** Interventional Oncology Unit with Integrate Section of Translational Medical Oncology, National Cancer Centre, Giovanni Paolo II of Bari, 70124 Bari, Italy; giroran@tiscali.it

“Angiogenesis and Anti-Angiogenesis in Oncology: From Bench to Translational Research and Bedside Investigation”. This Special Issue of *International Journal of Molecular Sciences* focuses on the above topic. From a historical point of view, in 1971 Judah Folkman postulated the concept that angiogenesis is an essential process for tumor growth and development. Dr. Folkman also suggested the hypothesis to block tumor growth by inhibiting angiogenesis. At that time, the angiogenic pathways were unknown, and no anti-angiogenic drugs were available. Following studies clarified that angiogenesis is the process of new blood vessels formation from a pre-existing vascular bed and is a fine-regulated pathway sustained by a plethora of pro-angiogenic and anti-angiogenic factors. Among them, the most important classical factor is the vascular endothelial growth factor (VEGF) that was characterized by Napoleone Ferrara in 1989. More recently, it has been well demonstrated that pro-angiogenic factors can be secreted by tumor cells and in particular by inflammatory cells such us macrophages and mast cells. In this context, non-classical pro-angiogenic factors such as tryptase have been found, and tyrosine kinase receptors involved in the angiogenic process have been characterized. Molecular pathways sustained by pro-angiogenic factors overexpression in the tumor microenvironment have been elucidated, and, in particular, tumor hypoxia has been identified as the main driver of angiogenesis. Hypoxia acts by increasing the expression of hypoxia-inducible factors 1 and 2, which in turn are able to activate the transcription of genes codifying VEGF proteins and other pro-angiogenic polypeptides. Pro-angiogenic factors, their specific tyrosine kinase receptors, and tyrosine kinase receptors that activate cells (e.g., c-Kit receptor) able to secrete pro-angiogenic factors are potential targets of angiogenic inhibitors. Bevacizumab, a murine monoclonal humanized antibody blocking VEGF, is the first approved antiangiogenetic drug and the most widely used in oncology.

The original researches and review papers published in this Special Issue of *International Journal of Molecular Sciences* journal summarize the most recent knowledge regarding the molecular and cellular pathways involved in the tumor angiogenic process and the main individuated targets to inhibit this process. Approved and experimental drugs targeting angiogenesis are discussed from a biological and a clinical point of view. All manuscripts published in the Special Issue have been planned starting from the bench and translating the scientific data to the patient bedside.

Here, the following five published research manuscripts are presented. Dr. Sammarco and co-workers present a research paper regarding tumor-associated macrophages, mast cells positive to tryptase, and their correlation with angiogenesis in surgically treated gastric cancer patients. In this work, the angiogenic pathways induced by macrophages and mast cells are discussed, and a hypothesis to inhibit these pathways is proposed [1]. Dr. de Souza Junior and co-workers focus on mast cells, demonstrating in an in vitro model the role of this cell type in stimulating endothelial cells proliferation and angiogenesis. The angiogenic factors induced by mast cells are analyzed. This study supports the central role of mast cells in tumor angiogenesis [2]. My co-workers and I carry out a trial employing several Bevacizumab-based schedules together with integrated regional deep capacitive hyperthermia in metastatic cancer patients. Both the cellular and the molecular antiangiogenic effects of hyperthermia and the additive effect of Bevacizumab are discussed [3]. Dr. Burger and co-workers propose a study in which Bevacizumab is utilized alone or in combination with several chemotherapies to treat patients affected by primary brain tumors including glioblastomas. The biological basis of the infiltrative growth of brain malignancies supported by angiogenesis is examined, and the rationale to use Bevacizumab is analyzed. Clinical results of the study are shown [4]. Dr. Rocha and co-workers study A3 adenosine receptor inhibition and the following effect on vasculogenesis mediated by glioblastoma stem-like cells under hypoxia in an in vivo glioblastoma tumor model. Endothelial markers are evaluated at the cellular and molecular level. The results of this research suggest that the inhibition of glioblastoma stem-like cells and of the adenosine axis may be a therapeutic target for glioblastoma [5].

Beside the above research papers, the following ten review manuscripts are here presented. Dr. Tamma and Dr. Ribatti focus on the role of bone marrow niches in the control of the fate of hematopoietic stem cells (HSCs) and present the role of the bone marrow (BM) niches in the regulation of vasculogenesis and angiogenesis. The authors summarize the alterations of the signals in the niche microenvironment that are involved in many aspects of tumor progression and vascularization. In this work, it is suggested that further knowledge could provide the basis for the development of new therapeutic strategies. This review focuses on the description of the role of BM niches in the control of the fate of HSCs and also highlights the role of the BM niches in the regulation of vasculogenesis and angiogenesis. Moreover, alterations of the signals in niche microenvironment are involved in many aspects of tumor progression and vascularization and further knowledge could provide the basis for the development of new therapeutic strategies. the A3 adenosine receptor [6]. Dr. Nienhüser and Dr. Schmidt analyze the most important angiogenic factors and their effects in gastric cancer patients and then highlight clinical trials including anti-angiogenic drugs. Potential biomarkers predictive of a tumor response to anti-angiogenic therapy are presented [7]. Dr. Angelucci and co-workers show the molecular mechanisms that determine tumor-associated microvessel formation in colorectal cancer (CRC). discuss the potential role of angiogenic factors as diagnostic, prognostic and predictive biomarkers in CRC. The potential role of angiogenic factors as diagnostic, prognostic, and predictive biomarkers in colorectal cancer is explored [8]. To this regard, it is interesting to comment that Bevacizumab-based chemotherapy may decrease the frequency of granulocytic myeloid-derived suppressor cells and increase that of pro-inflammatory helper T cells, rendering the tumor microenvironment favorable for immune checkpoint inhibitor treatment. On these bases, the combination of immunotherapy and anti-angiogenic drugs for colon cancer will be a very interesting research field in the future. Dr. Kareva presents the combination of immune checkpoints inhibitors with metronomic chemotherapy and the role of microenvironment modulation in targeting resistant cancer cells. The influence of microenvironments in tumor angiogenesis is discussed [9]. Dr. Loizzi and co-workers focus on the pathways of neovascularization in ovarian cancer. The most recent clinical trials based on Bevacizumab plus chemotherapy are highlighted [10]. Dr. Manzo and co-workers summarize the molecular mechanisms of angiogenesis in non-small cell lung cancer. In this context, Bevacizumab and the more recent anti-angiogenic drugs Ramucirumab and Nintendanib are presented in terms of mechanism of action and clinical outcome [11]. Dr. Kong and co-workers revise the main anti-angiogenic targets for approved and novel experimental monoclonal antibodies. Data for VEGF and VEGF receptor axis, platelet derived growth factor (PDGF) and PDGF receptor axis, and Angiopoietin (Ang) 1-2 and Ang 1-2 receptor axis are summarized [12]. Dr. Bendtsen and co-workers focus on hypertension as the main side effect caused by Lenvatinib and Everolimus in the treatment of metastatic renal cell carcinoma. The molecular mechanisms of VEGF and VEGF receptor targeting by Lenvatinib and Everolimus are discussed [13]. Dr. Randrup Hansen and co-workers put in evidence the effects and side effects of Sorafenib and Sunitinib in the treatment of metastatic renal cell carcinoma. Among the side effects, hypertension is again the most relevant and it is widely discussed [14]. Finally, Dr. Niccoli Asabella and co-workers overview the status of multimodality imaging in tumor angiogenesis. The criteria of tumor response to anti-angiogenic drugs based on positron emission tomography metabolism are analyzed [15].

The present status and future directions of angiogenesis and anti-angiogenesis research are the focus of all published papers.
